# Out-of-Hospital Cardiac Arrest: Public-Access Defibrillation and System Approaches to Minimize Avoidable Delay

**DOI:** 10.3390/jcm15062141

**Published:** 2026-03-11

**Authors:** Gianluca Pagnoni, Maria Giulia Bolognesi, Serena Bricoli, Luca Rossi, Allegra Arata, Daniela Aschieri

**Affiliations:** Cardiology Unit, Specialistic Medicine Department, Guglielmo da Saliceto Hospital, 29121 Piacenza, Italy; m.bolognesi@ausl.pc.it (M.G.B.);

**Keywords:** out-of-hospital cardiac arrest (OHCA), automated external defibrillator (AED), public-access defibrillation (PAD), time-to-shock, citizen responder, first responder, AED registry, mobile applications, drone-delivered AED, neurological outcome

## Abstract

Out-of-hospital cardiac arrest (OHCA) remains a leading cause of sudden death worldwide, with wide variation in reported incidence and outcomes driven by heterogeneity in registries, emergency medical services (EMS) organization, and case definitions. Despite substantial advances in resuscitation systems, survival after EMS-treated OHCA generally remains below 10%, and outcomes are critically time dependent. Delays in emergency call activation, bystander cardiopulmonary resuscitation (CPR), and—most importantly—early defibrillation are associated with a rapid decline in return of spontaneous circulation and favorable neurological recovery. This narrative review synthesizes current evidence and implementation strategies aimed at reducing “time-to-CPR” and “time-to-shock,” with a specific focus on public-access defibrillation (PAD) as a tool to mitigate avoidable delay. Randomized trials and large registry studies consistently demonstrate that automated external defibrillator (AED) use before EMS arrival is a key determinant of survival in patients with shockable rhythms. However, the real-world effectiveness of PAD remains limited by suboptimal AED placement, restricted 24/7 accessibility, low public awareness, and underutilization driven by fear and lack of confidence. We compare different PAD delivery models—including EMS-based, police and first-responder-based, and fully integrated community systems—and summarize evidence supporting targeted, high-yield AED deployment and cost-effectiveness. In addition, we review emerging strategies to reduce avoidable delay and strengthen the early links of the chain of survival, such as school-based training programs, smartphone- and SMS-based citizen-responder networks, improved dispatch recognition of cardiac arrest (including artificial intelligence–supported tools), and drone-enabled AED delivery. Across these approaches, patient benefit critically depends on system integration, alert performance, and true AED accessibility. Finally, we describe the Italian “Progetto Vita” experience as a community-integrated model explicitly designed to minimize avoidable delay through widespread AED deployment, lay responder training, and real-time integration with EMS. We conclude by outlining future priorities, including the development of robust national OHCA registries and scalable solutions for the high burden of cardiac arrests occurring at home, such as population-level deployment of low-cost, ultra-portable AEDs.

## 1. Introduction

Out-of-hospital cardiac arrest (OHCA) remains a major public health challenge and a leading cause of sudden death worldwide. Above all, OHCA prognosis is time dependent: early recognition, immediate bystander cardiopulmonary resuscitation (CPR), and rapid defibrillation for shockable rhythms are the key determinants of return of spontaneous circulation and favorable neurological recovery.

In this context, public-access defibrillation (PAD) represents one of the most impactful strategies to reduce collapse-to-shock time and bridge the gap before EMS arrival. However, real-world PAD effectiveness is frequently constrained by limited 24/7 AED accessibility, mismatch between device placement and arrest location, and persistent underutilization. Alongside conventional PAD, several system-level innovations—citizen responder activation, enhanced dispatch recognition (including AI-assisted tools), and drone-enabled AED delivery—have emerged with the shared goal of reducing avoidable delay and improving early defibrillation in the community.

This review summarizes contemporary evidence and organizational models aimed at shortening “time-to-CPR” and “time-to-shock,” discusses barriers and cost-effectiveness considerations, and highlights future directions to expand equitable access to early defibrillation, with particular attention to residential OHCA and scalable community-integrated programs. Throughout the manuscript, we specify the time origin for each metric: collapse-to-CPR/shock (from witnessed collapse/recognition when available) for physiological/PAD effects, and call receipt-to-CPR/shock (from emergency call receipt) for dispatch-based KPIs and studies relying on dispatch logs.

### 1.1. Methods: Literature Search and Selection

This manuscript is a targeted narrative review aimed at summarizing contemporary evidence and implementation models designed to reduce “time-to-CPR” and “time-to-shock” in adult out-of-hospital cardiac arrest (OHCA), with a focus on public-access defibrillation (PAD) and system approaches to minimize avoidable delay.

We performed a targeted narrative literature search in PubMed/MEDLINE and Embase (supplemented by Scopus/Web of Science when needed) from 1 January 2000 through 31 December 2025. Search terms combined controlled vocabulary and free text related to OHCA and early defibrillation, including “out-of-hospital cardiac arrest/OHCA”, “public-access defibrillation/PAD”, “automated external defibrillator/AED”, “bystander defibrillation”, “citizen responder/first responder”, “smartphone alert”, “AED registry”, “dispatch-assisted CPR”, “dispatch recognition”, and “drone-delivered AED”.

Eligibility focused on adult OHCA and community response systems, including studies on PAD/AED accessibility and deployment, first-responder or citizen-responder activation (SMS/app-based), dispatch-assisted CPR and dispatch recognition tools (including AI-supported approaches), AED registries and integration with dispatch, and drone-enabled AED delivery. We prioritized randomized trials (when available), large registry/observational studies, systematic reviews/meta-analyses, health economic evaluations, and international guideline/consensus documents. We excluded studies primarily focused on in-hospital cardiac arrest, pediatric populations, animal studies, and reports not directly informing early defibrillation or pre-hospital system performance.

Evidence was complemented by manual screening of reference lists and key position papers. Given the narrative scope and heterogeneity of designs and endpoints, no formal meta-analysis or pooled effect estimates were performed; findings were synthesized qualitatively with emphasis on determinants of process measures (e.g., bystander CPR/AED use, time-to-shock) and patient-important outcomes (e.g., survival and neurological recovery).

AI-Assisted Editing Statement. During manuscript preparation, ChatGPT (OpenAI) 5.2 was used solely for language editing and stylistic refinement (grammar, clarity, and readability) and to improve the presentation of visual material. All AI-assisted output was critically reviewed and approved by the authors, who take full responsibility for the final content of the manuscript.

### 1.2. Epidemiology of Out-of-Hospital Cardiac Arrest (OHCA)

According to the second report from the International Liaison Committee on Resuscitation (ILCOR), the incidence of EMS-treated OHCA across countries in North America, Europe, Asia, and Oceania ranges from 28 to 244 per 100,000 population, with marked regional variation [1].

In the United States, CARES data report an incidence of EMS-treated OHCA of 89 per 100,000 population across all ages, with substantial state-level variability (49.9–137 per 100,000) [2]. In EuReCa TWO, the incidence of EMS-attempted resuscitation in Europe was 56 per 100,000 inhabitants (26–91 per 100,000), with country-specific estimates ranging from 19 per 100,000 in Spain to 97 per 100,000 in Poland, and an increasing trend over recent years [3]. Reported incidence appears lower in parts of Asia (25.5 per 100,000 per year), whereas in Australia it has been reported as 107 per 100,000 inhabitants annually. In a meta-analysis of Italian studies, the incidence of OHCA with attempted resuscitation was estimated at 55 per 100,000 population per year [4].

OHCA incidence increases with age and is higher in males than in females; women tend to experience OHCA at an older age and often in the presence of cardiovascular risk factors. Environmental factors may also contribute, with some data suggesting an association between OHCA and daily levels of particulate air pollution [5,6,7].

Most OHCAs occur at home or in private residences, followed by public locations; temporal patterns have also been reported, with higher incidence in specific time windows and seasonal peaks (including December) in some studies [2,8].

Historically, OHCA etiology has been categorized as medical (cardiac, respiratory, anaphylaxis, gastrointestinal bleeding, unknown) versus non-medical (trauma, drug overdose, asphyxia, drowning). In adults, ischemic heart disease accounts for the majority (~80%) of cardiac sudden cardiac death, followed by cardiomyopathies, whereas channelopathies predominate in younger individuals [9].

Despite advances in prehospital care and cardiopulmonary resuscitation, survival after OHCA remains generally below 10%, with wide variability across countries. This heterogeneity reflects differences in EMS activation criteria, prehospital organization, and national legislative frameworks, making direct comparisons between registries challenging. In CARES, survival after EMS-treated OHCA was 9.3% (range 5.5–15.4%), while in EuReCa ONE, survival was 8% (range 0–18%), with lower rates in the Middle East (2–8%) [1,2,10]. Survival depends on multiple factors, including early resuscitation attempts, sex, etiology, initial rhythm, pre-existing comorbidities, location of arrest, and socioeconomic determinants.

### 1.3. Determinants of Survival: Time-to-Shock, Time-to-CPR, and Avoidable Delay

In OHCA, time is a key determinant of survival and neurological recovery. In this section, unless otherwise specified, “time-to-CPR” and “time-to-shock” refer to collapse-to-first chest compression and collapse-to-first shock (when collapse/recognition time is available). Cellular injury begins within 60–90 s after circulatory arrest, and irreversible cerebral injury accelerates after approximately 4 min without perfusion in the absence of immediate CPR. The “chain of survival,” first described in 1991, emphasizes a sequence of time-sensitive actions to maximize survival: (1) early access/EMS activation, (2) early basic life support to slow deterioration, (3) early defibrillation to restore a perfusing rhythm, and (4) early advanced life support and stabilization [11].

More recently, American guidelines updated this framework, and in 2020 two additional links were introduced to highlight post-resuscitation care and increased emphasis was placed on early recognition and engagement of lay responders [12,13].

From an implementation perspective, these links can be reframed as opportunities to reduce avoidable delay—the modifiable time lost before effective CPR and defibrillation are delivered. Avoidable delay typically includes (i) delayed recognition and delayed emergency call activation, (ii) delayed initiation of bystander CPR, and (iii) delayed AED retrieval and shock delivery when a shockable rhythm is present. These components are measurable and actionable, and they complement conventional EMS response metrics by focusing on what happens before EMS arrival.

Early initiation of resuscitative interventions—particularly before EMS arrival—has been consistently associated with better outcomes. Bystander CPR and public AED use improve survival and neurological outcome by maintaining perfusion and increasing the likelihood of successful defibrillation in shockable rhythms [14,15]. Using the U.S. CARES registry, Nguyen et al. analyzed 78,048 witnessed OHCAs treated with lay bystander CPR (2013–2021) and reported a median call receipt-to-bystander CPR time 2 (IQR 1–5) minutes; increasing delays in CPR initiation showed a graded inverse association with survival to hospital discharge and favorable neurological survival [16]. Similar patterns were observed in EuReCa TWO, where bystander CPR rates varied widely (13–82%) across participating countries [3].

Outcome is also strongly influenced by the presenting rhythm. Survival is higher when the initial rhythm is shockable (ventricular fibrillation [VF] or pulseless ventricular tachycardia [VT]), which are more common early after collapse, particularly in witnessed arrests. In an early study by Bayés de Luna, shockable rhythms were reported in ~85% of arrests in device-recipient patients, whereas in other series this proportion declined to ~60% after 4–8 min, consistent with time-dependent deterioration to non-shockable rhythms (e.g., asystole), which carry a poorer prognosis [17].

Accordingly, survival decreases rapidly with each minute of delay to defibrillation. Stieglis et al. showed that the probability of VF termination declined from 93% when the emergency call-to-first shock interval was <6 min to 75% when it exceeded 16 min [18]. Public deployment of AEDs and bystander intervention can shorten time-to-shock. In a prospective study from the Resuscitation Outcomes Consortium (ROC), bystander AED use was associated with improved overall outcomes and neurological status. The 2015 ERC guidelines emphasized the critical importance of EMS–bystander interaction to support both CPR and AED use [19].

Key barriers that remain to be addressed include: (i) technologies that facilitate early OHCA recognition, (ii) rapid identification and true 24/7 accessibility of AEDs, (iii) education and awareness programs to promote a culture of defibrillation, (iv) strategies to reduce hesitation and perceived ethical concerns among bystanders, and (v) maintenance of high-quality registries in accordance with reporting guidelines.

## 2. Public Access Defibrillation

### 2.1. Evidence Supporting Public Access Defibrillation

One of the earliest and most influential demonstrations of PAD effectiveness was reported by Valenzuela and colleagues in the context of cardiac arrests occurring in Las Vegas casinos. The primary outcome was survival to hospital discharge, which was 74% when the first defibrillation was delivered ≤3 min after a witnessed collapse, compared with 49% when defibrillation occurred >3 min after collapse, underscoring the steep time-dependence of benefit [20].

The Public-Access Defibrillation (PAD) Trial, published in 2004, provided the strongest experimental evidence supporting PAD. In this community-based, cluster-randomized trial, units assigned to CPR plus AED training had more survivors to hospital discharge (30/128 arrests) than units assigned to CPR-only training (15/107 arrests; relative risk 2.0, 95% CI 1.07–3.77; *p* = 0.03), although only two survivors occurred in residential complexes [21].

In a large population-based evaluation from the Resuscitation Outcomes Consortium (ROC), application of an AED before EMS arrival was independently associated with a significantly greater likelihood of survival (adjusted OR 1.75, 95% CI 1.23–2.50; *p* < 0.002), supporting the effectiveness of PAD in real-world community settings when integrated into the chain of survival [14].

Finally, early defibrillation strategies combined with dual-dispatch systems have been explored in Scandinavia. In the SALSA pilot, the proportion of patients alive at 1 month increased from 4.4% to 6.8% (adjusted OR 1.6; 95% CI 0.9–2.9), and among witnessed arrests from 5.7% to 9.7% (adjusted OR 2.0; 95% CI 1.1–3.7), consistent with improved outcomes when first responders are dispatched in parallel with EMS [22].

The effectiveness of PAD depends not only on the number of AEDs deployed but also on their spatial and temporal accessibility.

Several registry-based studies have shown that increasing AED density improves the theoretical coverage of OHCA events. However, a substantial mismatch often exists between AED location and actual cardiac arrest sites. Many arrests occur within a short distance of an AED that is never retrieved or used, highlighting the importance of system design rather than simple device count [15,23].

One of the most significant limitations of current PAD programs is limited AED accessibility outside business hours. Studies have demonstrated that a large proportion of public AEDs are located inside buildings that are closed during evenings, nights, or weekends—periods when a considerable number of OHCAs occur. As a result, the proportion of cardiac arrests truly “covered” by an accessible AED is substantially lower than suggested by density alone [24].

Data consistently indicate that survival improves when AEDs are both nearby and accessible and when their use is actively integrated into emergency response systems. Registries, dispatcher guidance, and clear signage all contribute to translating AED availability into actual defibrillation and improved outcomes.

### 2.2. Organizational Models for Early Defibrillation

In EMS-based models, professional emergency services remain the primary providers of defibrillation. AEDs are deployed on ambulances and rapid response vehicles, and dispatchers provide telephone-assisted CPR to bystanders [19].

In police-based PAD models, law enforcement vehicles are equipped with AEDs and dispatched in parallel with EMS to suspected cardiac arrests [25]. Police patrols are often widely distributed and may be closer to the scene than EMS units. Studies from North America and Europe have shown that police AED programs are feasible and can significantly reduce time to first shock in selected settings.

Combined models represent the most comprehensive approach to early defibrillation. These systems integrate public AED deployment, first responders (police, firefighters, volunteers), dispatcher-assisted CPR, and increasingly, smartphone-based citizen responder activation.

Evidence from regions implementing dual dispatch and community responder systems suggests meaningful reductions in response times and improvements in survival, particularly for witnessed arrests.

Beyond clinical effectiveness, the sustainability of Public Access Defibrillation (PAD) programs depends on their economic impact. Multiple health economic evaluations conducted across different healthcare systems have consistently shown that PAD programs are cost-effective when compared with commonly accepted thresholds for healthcare interventions [26].

Early analyses following the implementation of large PAD programs in North America and Europe demonstrated that the incremental cost-effectiveness ratio (ICER) of PAD falls well within ranges considered acceptable in high-income countries. These findings are particularly robust in settings with a higher incidence of witnessed cardiac arrest and a higher prevalence of shockable rhythms, such as airports, transportation hubs, sports facilities, and densely populated urban areas [27].

The cost-effectiveness of PAD improves further when AEDs are strategically deployed and integrated into existing emergency response infrastructures, thereby limiting redundancy and maximizing device utilization [28].

Several modeling studies have demonstrated that PAD programs yield a substantial number of life-years gained per patient, reflecting the relatively young age and favorable neurological outcomes of many OHCA survivors. As a result, even modest improvements in survival translate into significant gains in long-term survival [29].

Analyses comparing PAD with CPR-only strategies consistently show that the additional cost per life-year gained is relatively low. In many studies, the ICER for PAD is comparable to, or more favorable than, that of other widely adopted cardiovascular interventions, including pharmacological therapies and implantable devices [30,31].

The economic value of PAD programs is strongly influenced by several key factors:

1. Incidence of OHCA and proportion of shockable rhythms

Higher event rates and higher proportions of VF/VT markedly improve cost-effectiveness.

2. Time to defibrillation

Programs that achieve meaningful reductions in time to first shock yield greater survival benefits and better economic returns.

3. AED accessibility and utilization rates

AEDs that are accessible 24/7 and frequently used generate a higher return on investment than devices placed in low-yield or poorly accessible locations.

4. Integration with existing systems

PAD programs embedded within EMS dispatch systems, first-responder networks, and digital AED registries tend to be more cost-effective than standalone initiatives.

## 3. Optimizing Early Defibrillation Beyond EMS: Lay Training, Digital Responder Networks, and Drone-Enabled AED Delivery

### 3.1. Layperson Response and Training Models

Public-access defibrillation (PAD) performed by laypersons is associated, in national and local registries, with higher survival and favorable neurological outcome among patients presenting with shockable rhythms, consistent with the time-dependent benefit of early defibrillation [32,33,34].

“Community-wide” programs integrating mass training, AED registries interoperable with dispatch centers, and promotion of lay response have shown temporal improvements in BLS rates and survival; however, disentangling the specific contribution of training from that of infrastructure and dispatch performance remains methodologically challenging [35].

Surveys assessing population knowledge and attitudes indicate that AED availability alone is insufficient: limited AED literacy, poor awareness of device location, and reluctance to intervene persist, and may be mitigated through integrated training (BLS + AED) and periodic refreshers [36,37].

Blended strategies (micro-learning, serious games, low-dose/high-frequency retraining) may improve performance and engagement, but require robust educational governance and standardization to reduce the risk of “perceived competence” not translating into real-world performance during emergencies [38].

In workplace settings, AED presence and use are variable and often not systematically monitored; the expected impact depends primarily on crowd density, internal response times, and retraining quality rather than on device placement alone [39].

### 3.2. Role of Technology and Apps in Early Defibrillation

“Citizen responder” networks activated via SMS/apps aim to reduce the no-flow/low-flow interval and increase the likelihood of AED delivery before emergency medical services (EMS); however, the literature is heterogeneous in activation radius, recruitment criteria, integration with AED registries, and feedback systems, and still includes relatively few studies with clinical outcomes [40,41].

Most evidence supporting smartphone/SMS citizen-responder systems comes from observational registries and implementation studies and is therefore vulnerable to residual confounding and selection mechanisms (e.g., witnessed status, urban density, EMS response time, and a higher likelihood of shockable rhythms when responders arrive early). As a result, improvements in process measures (CPR rates, AED retrieval, call receipt-to-first shock time) do not uniformly translate into patient-important outcomes (survival/neurological recovery) across settings. Key failure modes include limited alert specificity/actionability (false positives/cancellations), access barriers (building entry and true 24/7 AED availability), and insufficient probability of arrival-before-EMS.

In a randomized trial in Stockholm, the positioning/dispatch system was activated in 667 OHCAs and significantly increased bystander-initiated CPR before arrival of ambulance/fire/police from 48% (172/360) to 62% (188/305) (absolute difference 14 percentage points; *p* < 0.001). However, secondary outcomes (e.g., ROSC and 1-month survival) were not significantly different, illustrating that improved process metrics may not translate into hard endpoints in settings with short EMS response times and low shockable-rhythm prevalence [15].

In the HeartRunner implementation study (Denmark), citizen responders were alerted in 819 suspected OHCAs (438 confirmed), and responders arrived before EMS in 42% of cases. Arrival before EMS was associated with higher odds of bystander CPR (OR 1.76, 95% CI 1.07–2.91) and a >3-fold increase in bystander defibrillation (OR 3.73, 95% CI 2.04–6.84) [42].

In a binational Denmark–Sweden cohort (*n* = 1271; 81% occurring at home), at least one volunteer responder arrived before EMS in 37.0% of home arrests and 34.7% of public arrests. Bystander defibrillation was substantially higher when a volunteer arrived first both at home (15.5% vs. 2.2%, *p* < 0.001) and in public locations (32.1% vs. 19.6%, *p* = 0.030); among patients with shockable rhythm, the corresponding differences were 52.7% vs. 11.5% at home and 60.0% vs. 37.8% in public. Despite these process gains, no statistically significant improvement in 30-day survival was observed when volunteers arrived before EMS, underscoring residual confounding and the importance of pragmatic trials powered for patient-important outcomes [43].

For GoodSAM, registry-linked analyses reported overall survival to hospital discharge of 9.6% in London and 7.2% in East Midlands; however, alert acceptance occurred in only 1.3% and 5.4% of OHCAs, respectively. When an alert was accepted, survival to hospital discharge was higher (adjusted OR 3.15 [1.19–8.36] in London; 3.19 [1.17–8.73] in East Midlands), suggesting potential benefit when responders truly engage. Operational performance is strongly influenced by true travel time/distance: using real-world routing (vs. straight-line distance) increased estimated travel distance by a median ~200–300 m, changed the “nearest AED” in 26% of cases, and demonstrated that distance alone did not predict alert acceptance—supporting the need to optimize dispatch radius and alert design beyond Euclidean proximity [44,45].

For PulsePoint, user-level survey data highlight “actionability” limits: only 23% of notified users responded, 28% of responders did not reach the scene, and only 32% of those arriving encountered an actual presumed arrest (unconscious/not breathing normally). Among those who arrived before EMS and found a true arrest, 79% initiated CPR—suggesting high willingness when alerts are accurate and responders arrive in time [46].

In France, an SMS-based “citizen rescuer” system has been associated with higher bystander CPR rates and increased 30-day survival, although it remains vulnerable to selection bias (witnessed events, urban density, caller compliance) typical of community-based programs. In Italy, DAE RespondER represents a regional integration model with the EMS dispatch center; however, large-scale surveys indicate that logistical barriers (AED availability at the time of alert, site accessibility) and cognitive-emotional factors substantially influence acceptance and response [47,48].

Regarding safety and sustainability, the psychological impact on volunteers is, on average, limited but not negligible, with higher-risk subgroups (lack of training, younger age, direct involvement in CPR), supporting structured debriefing and support within mature programs [49].

Artificial intelligence applied to dispatch (OHCA recognition during emergency calls) is promising for improving sensitivity and shortening time to diagnosis; however, translation into clinical benefit requires workflow integration, error management, and prospective validation. In a randomized study, a machine-learning dispatcher support tool reduced call receipt-to-dispatcher OHCA recognition time, suggesting that this interval is necessary but insufficient unless it translates into higher rates of effective CPR/AED use on scene [50,51].

AI-enabled dispatch should therefore be viewed as an enabling layer rather than a stand-alone solution; clinical impact depends on workflow integration, error/false-alarm management, local prospective validation, and sustained downstream effects on CPR and AED application.

Digital technologies “beyond dispatch” (e.g., contactless detection and consumer smart devices) expand the innovation landscape but remain far from routine implementation and raise concerns regarding privacy, reliability, and potential inequities in access [52].

### 3.3. Drones for AED Delivery

Drone delivery primarily targets avoidable delay due to geographic and operational constraints, but its clinical value ultimately depends on human factors (continued CPR, willingness to apply the AED) and dispatch precision.

GIS analyses and simulation studies suggest that drones may precede EMS predominantly in rural settings or where EMS response times are prolonged; in urban environments, the average benefit tends to be smaller and is highly dependent on base-network design and regulatory/weather constraints [53,54].

In early real-world evidence from Sweden, drone-based AED delivery for suspected OHCA was feasible and safe, with high successful delivery rates and a clinically meaningful median time advantage when drones arrived before ambulances [55,56].

Nevertheless, even when an AED is delivered before EMS, the proportion of cases in which the device is actually applied to the patient may remain low due to human factors (maintaining CPR, hesitation, access to the drop-off point) and the proportion of suspected OHCAs that are cancelled as false alarms [57].

Evidence for drone-delivered AEDs is strongest for feasibility and time advantage, whereas direct evidence for improved survival/neurological outcome remains limited. The time gain can be negated by operational constraints (weather, no-fly zones, night operations) and “last-meter” human factors (CPR interruption, difficulty retrieving/using the AED). Therefore, drones are most plausibly beneficial where geography or prolonged EMS response creates a consistent and meaningful time gap.

Human–drone interaction and mixed-methods studies suggest that bystanders perceive drone delivery as acceptable and potentially useful, yet highlight operational challenges (drone/AED identification, instructions, risk of CPR interruption) that require targeted training and dedicated dispatch protocols [58,59].

In Canada, published evidence is largely limited to feasibility in simulated scenarios and community acceptability (e.g., Caledon), indicating that population-level “AED literacy” is a key prerequisite and potentially more determinant than delivery technology itself [60].

In the United States, the literature mainly includes reports/letters and simulation studies, alongside implementation- and cost-oriented reviews; therefore, direct clinical evidence (real dispatch for true OHCA with patient-important endpoints) remains limited relative to perceived technological maturity [46].

Major barriers to real-time integration include aviation regulation, risk management, no-fly zones, night operations, weather, legal liability, and interoperability with CAD/dispatch systems; studies focused on night scenarios confirm feasibility but also increased operational complexity [61].

Advanced machine-learning–based dispatch models may reduce unnecessary flights while preserving time gains, but require local datasets, continuous monitoring, and auditing to prevent drift and systematic bias.

Overall, drones may act as a “force multiplier,” particularly where the chain of survival is constrained by prolonged EMS times and low AED density/accessibility; however, the expected impact depends on integration with lay training, citizen responder networks, and dispatch quality rather than on the delivery vector alone (Table 1).

### 3.4. Comparative Summary and Strength of Evidence

PAD has the strongest outcome-level evidence (including randomized and large-registry data) when AEDs are strategically placed, truly accessible 24/7, and integrated with dispatch. Training and retraining interventions primarily improve readiness and process performance, with outcome effects that are often context-dependent and intertwined with system infrastructure. In contrast, smartphone/SMS citizen-responder systems and AI-enabled dispatch are supported largely by implementation/observational evidence showing improvements in process metrics (CPR, AED-on-scene, time-to-shock), whereas drones remain supported mainly by feasibility and simulation studies with variable “last-meter” human factors.

Recent ILCOR CoSTR 2025 and ERC Guidelines 2025 reinforce the central message of this review: improving outcomes in shockable OHCA depends on minimizing avoidable delay through early recognition, prompt EMS activation, high-quality bystander CPR, and rapid AED use. The ERC 2025 ‘Systems Saving Lives’ recommendations further support system integration by advocating first-responder programmes with dispatch notification of nearby registered responders (including to private residences) and by recommending dispatcher-assisted public-access AED systems linked to AED registries, while discouraging locked or inaccessible AED cabinets. In line with this framework, we emphasize that PAD effectiveness depends less on the absolute number of devices and more on strategic placement, true 24/7 accessibility, and dispatch-enabled activation. Conversely, the ERC 2025 guidance highlights that AI and other digital health technologies show potential but are not yet ready for routine use; accordingly, we present AI-supported dispatch and other emerging layers primarily as investigational strategies requiring prospective evaluation, governance, and equity safeguards [62,63,64,65].

Ultra-portable/personal-use AEDs represent a promising concept to address the residential gap, but require pragmatic evaluation of usability, safety, cost-effectiveness, and patient-important outcomes.

### 3.5. Generalizability, Equity, and Scalability

Implementation of PAD and technology-enabled responder systems is strongly conditioned by local resources and EMS maturity. In low- and middle-income settings, limited dispatch coverage, lower AED availability, and competing health priorities may shift the focus toward scalable fundamentals—community CPR training, dispatcher-assisted CPR, reliable emergency access, and targeted AED placement in high-yield public sites—before adopting technology-intensive layers. Socioeconomic disadvantage can further reduce AED availability and bystander response (e.g., residential crowding, building access barriers, lower AED literacy), suggesting that equity-oriented placement policies, public education, and open AED mapping are needed to avoid widening disparities. Finally, interventions should be viewed as modular: registries/AED mapping and dispatch-assisted CPR are broadly scalable, whereas app-based activation, AI support, and drone delivery require higher levels of dispatch integration, data governance, and operational capacity to deliver consistent benefit [1,3,30].

## 4. International and Italian Experiences: Progetto Vita and National Models

### 4.1. The Italian Experience: Progetto Vita and National Models

Progetto Vita was founded in Piacenza in 1998, thanks to the idea of Professor Alessandro Capucci to develop a rapid intervention system with AEDs (which were not yet available on the European market) to improve the survival of patients suffering from OHCA (Out-of-hospital Cardiac Arrest). As the first such experience, it led to the introduction of AED training courses in Piacenza, even before these courses were included in Basic Life Support (BLS) training by scientific societies. The project set three priority goals that enabled its realization and the achievement of its results:

- Implementation and spread of AEDs across the entire urban and provincial area, inhabited by approximately 300,000 people.
- Raising awareness among the population and promoting the culture of early defibrillation through informative and motivational courses on the use of AEDs, accompanied, when necessary, by BLS-D courses.
- Integration of AEDs into the local emergency system (118). The implementation of the “Blue Code” demonstrates how the community has been involved in early defibrillation and in fostering a culture of emergency response.

In practice, the main implementation levers act on two proximate process outcomes: bystander CPR and pre-EMS AED utilisation. Training and public engagement primarily increase CPR initiation; AED placement, signage and true 24/7 access increase AED availability on scene; dispatch integration (AED registry + protocols) and first/citizen responder activation increase the likelihood that CPR and an AED are delivered before EMS, collectively reducing avoidable delay (recognition → CPR → shock).

The first results published by our group in Circulation in 2002 were subsequently confirmed by updated data through 2016 and 2025 [33,66,67].

For clarity, the PV-group includes OHCA events in which a trained lay responder (first responders/bystanders/law enforcement) arrived before EMS and performed the first AED rhythm analysis (and shock if indicated) prior to EMS arrival. The EMS group includes EMS-treated OHCA events in which EMS was the first team to reach the patient and provide rhythm analysis/defibrillation, i.e., no lay responder intervention occurred before EMS arrival.

Out of a total of 6996 OHCA cases in the Piacenza area, in 156 cases the intervention by laypersons (first responders, bystanders, or law enforcement) preceded that of EMS personnel (5.7 vs. 10.2 min, *p* < 0.001), showing a significantly higher percentage of shockable rhythms (69.9% in the Progetto Vita group vs. 10.9% in the EMS group, *p* < 0.001). These large crude differences should not be interpreted as causal effects of the responder type alone. They are likely influenced by selection/confounding mechanisms, including earlier arrival (capturing a higher proportion of VF/VT before rhythm deterioration), a higher likelihood of witnessed arrests, differences in location (public vs. residential), and other arrest characteristics that increase the probability of shockable rhythm and survival. Survival on arrival at the hospital was in fact 55.8% in patients assisted by lay responders and 15.6% in patients assisted by EMS healthcare personnel, with better survival to hospital discharge in cases treated by laypersons compared with EMS (46.2% vs. 2.9%, *p* < 0.001). Accordingly, the PV-group vs. EMS-group comparison should be considered observational and non-randomized. By definition, PV-group cases required that a lay responder arrived before EMS, which enriches the cohort for time-favorable scenarios and may correlate with witnessed status, public location, dispatcher recognition, and immediate access to an AED. Residual confounding is therefore expected even after adjustment, and the magnitude of crude group differences should be interpreted primarily as reflecting time advantage and case-mix differences. Long-term survival was also better in cases treated by lay responders when variability in response time, sex, location of cardiac arrest, and the presence of a defibrillable rhythm were taken into account. Over the study period, the increasing number of AEDs in fixed public places coincided with a substantial rise in ventricular fibrillation survival in the PV-group patients (22% for 2003–2012 to 73% for 2013–2022, *p* < 0.001). In multivariable logistic regression with mortality as the dependent outcome, each 1 min increase in call-to-arrival time was associated with higher odds of death (OR = 1.08 per minute), whereas PV-group first response (lay responder arrival before EMS) was associated with lower odds of death (OR = 0.28), after accounting for response time, sex, arrest location, and shockable rhythm. Because the model outcome is mortality, OR < 1 indicates lower mortality (higher survival) and OR > 1 indicates higher mortality. In a specific setting such as OHCA during sports activities, data from our experience are even more striking: a 93% survival rate in sports facilities equipped with AEDs, markedly higher than the 9% survival rate in sports facilities without AEDs [68].

### 4.2. Future Directions in Defibrillation Strategies

In the context of OHCA and early defibrillation, it is essential to focus the efforts of the cardiology scientific community. A key priority is the creation of a national cardiac arrest registry. Such a registry would allow a better understanding of organizational heterogeneity across the country and would enable a more precise definition of intervention and improvement strategies. At present, available data derived from a recent meta-analysis cover only 52% of the Italian population, with an asymmetric distribution largely originating from registries in Northern Italy. Nevertheless, despite these limitations, the data confirm a significant improvement in survival when the cardiac rhythm at the time of first intervention is defibrillable, with an overall survival of 10% for non-defibrillable rhythms and 25% for defibrillable rhythms. It is also important to note that Italy is below the European average both in terms of the proportion of people who perform CPR (26%) and in the use of AEDs (3.2%).

In the future, it would be desirable to adopt lower-cost technologies capable of delivering a more limited number of shocks, using less expensive devices that can be more easily disseminated, including for home use. Technologies are already under development aimed at miniaturizing AEDs—the so-called ultraportable AEDs—making them truly portable for everyday life, while still ensuring a “bridge” function until advanced medical services arrive, and at the same time allowing for wider and more capillary distribution within the community.

This paves the way for a shift from AED deployment in public spaces to the availability of AEDs in domestic settings for personal use. New-generation AEDs are expected to have “disposable” characteristics: pocket-sized, lightweight, compact, and low-cost.

Personal-use, single-use AEDs are intended to be lower-cost and easier to disseminate, and may increase availability of early defibrillation—particularly in home settings—by potentially reducing barriers related to device access and portability. However, whether these strategies translate into improved survival and neurological outcome remains unproven, and current evidence largely includes feasibility/implementation work and trial protocols (e.g., ultraportable defibrillator responder trials) [69].

At present, however, ultra-portable/personal-use AED strategies should be considered investigational, as evidence is still emerging and definitive effects on patient-important outcomes will require pragmatic trials and real-world evaluation of usability, safety, cost, maintenance, and equitable access—particularly in home settings.

Key implementation questions include: (i) cost and cost-effectiveness versus targeted PAD expansion and dispatch-enabled responder models; (ii) training strategy and refreshers (to avoid low use despite availability); (iii) maintenance logistics (battery/electrode replacement, expiry, readiness checks) and governance; (iv) equity and access (risk of widening disparities if uptake is driven by individual purchasing power); and (v) safety/human factors (ease of use under stress, avoidance of CPR interruptions, appropriate integration with dispatch guidance) [70,71].

Twenty-five years after the launch of Progetto Vita, we can conclude that the key to its success has been the simplicity of the approach to AED use. The primary objective was to ensure rapid defibrillation in case of OHCA by involving non-specialized individuals who are nevertheless informed about AED use. This result would have been difficult to achieve through the exclusive adoption of BLS-D courses, due to the shortage of professional staff and specialized instructors, as well as the associated costs. While recognizing the importance of BLS-D courses, these may represent a barrier to the widespread dissemination of early defibrillation, whose success depends on the broad dissemination of information, motivation, and engagement of the population that is not specifically trained in emergency care.

## 5. Conclusions

Early defibrillation remains the most time-critical intervention to improve survival and neurological outcomes in OHCA with shockable rhythms. The clinical value of public-access defibrillation (PAD) is primarily explained by shortening collapse-to-shock time; consequently, PAD performance depends less on the absolute number of deployed AEDs and more on strategic placement, true 24/7 accessibility, rapid identification of device location, and effective dispatch-enabled activation.

From a system perspective, these elements converge on a single actionable objective: reducing avoidable delay. Among currently available strategies, public-access defibrillation (PAD)—particularly when an AED shock is delivered before EMS arrival in shockable OHCA—shows the most consistent association with improved survival and favorable neurological outcome, especially when AED placement, true 24/7 accessibility, and dispatch/AED-registry integration are optimized. In parallel, integrated programs linking AED registries to EMS dispatch and activating first- and citizen-responder networks generally improve process indicators (AED retrieval, time-to-first shock) and are frequently associated with better outcomes in observational registries; however, heterogeneity across EMS systems and selection mechanisms (including a higher likelihood of shockable rhythms when responders arrive early) requires cautious interpretation. Newer approaches such as AI-supported dispatch and drone-delivered AEDs currently rest mainly on process, feasibility, and simulation evidence, and their incremental benefit is context-dependent, shaped by workflow integration, operational constraints, and “last-meter” human factors.

A persistent and high-impact gap remains in the residential setting, where most OHCAs occur and where AED access, building entry, and device availability represent major bottlenecks. Future implementation should prioritize coverage of home and near-home arrests through data-driven placement strategies, guaranteed accessibility, community engagement, and continuous quality improvement using measurable indicators (recognition-to-call, time-to-CPR, time-to-shock, bystander AED use, survival, and neurological outcome). Overall, PAD should be implemented as a coordinated system of care explicitly designed to minimize avoidable delay and deliver the earliest possible shock reliably across real-world scenarios.

## Figures and Tables

**Table 1:** Summary of the main strategies to strengthen early defibrillation across the pre-hospital chain of survival. The table contrasts (i) layperson PAD and training models, (ii) smartphone/SMS–activated citizen responder systems and AI-assisted dispatch, and (iii) drone-enabled AED delivery, by outlining the underlying mechanism (time-to-shock reduction), the predominant evidence signal (mostly process endpoints vs. patient-important outcomes), major sources of heterogeneity (AED accessibility—especially at home—alert specificity/actionability, probability of responder arrival before EMS, and regulatory/weather constraints), and the settings in which each approach is most likely to provide incremental benefit (longer EMS response times, low AED density/access, and high-quality integration with dispatch, AED registries, and retraining programs).

Component	Core Mechanism	What the Evidence Mainly Supports	Main Bottleneck (Why Results Vary)	Where It Likely Adds Most Value
Lay response & training (PAD + education)	Reduce collapse-to-shock by enabling bystander AED use and sustained readiness.	Outcome-level: AED shock before EMS in shockable OHCA → higher survival/neurology. Training/retraining mainly improves readiness and process performance.	24/7 accessibility and arrest–AED mismatch; low AED literacy/hesitation; limited home coverage.	High-yield public sites + schools as training multipliers; best with dispatch guidance and AED registry integration.
Apps/citizen responders + AI dispatch	Increase probability of CPR/AED before EMS via mobilization + faster OHCA recognition	Mostly observational/implementation evidence showing improvement in process endpoints (CPR, AED-on-scene/defibrillation) when responders arrive pre-EMS; AI tools may reduce call receipt-to-dispatcher OHCA recognition time, but patient-outcome benefit remains inconsistent	Alert specificity/false alarms; building access; responder arrival-before-EMS probability; variable registry/dispatch integration.	Longer EMS times; dense responder pool; reliable AED registry + dispatch activation; continuous QI feedback.
Drones for AED delivery	Deliver AED earlier than EMS, especially where geography delays response	Predominantly feasibility/observational and simulation evidence showing potential time advantage (largest in rural/long-EMS contexts); definitive survival/neurological benefit remains uncertain and context-dependent	Regulation/no-fly/weather/night ops; CAD integration; cancellations; last-meter human factors (CPR interruption, locating/using AED).	Rural/remote gaps; add-on to dispatch + citizen responders + training with clear drop-off and instruction protocols.

## Data Availability

No new data were created in this study.
